# A pilot study on non-invasive in situ detection of phytochemicals and plant endogenous status using fiber optic infrared spectroscopy

**DOI:** 10.1038/s41598-023-48426-5

**Published:** 2023-12-14

**Authors:** Shuyan Zhang, Randall Ang Jie, Mark Ju Teng Teo, Valerie Teo Xinhui, Sally Shuxian Koh, Javier Jingheng Tan, Daisuke Urano, U. S. Dinish, Malini Olivo

**Affiliations:** 1 Institute of Materials Research and Engineering, Agency for Science, Technology and Research (A*STAR), 2 Fusionopolis Way, Innovis #08-03, Singapore, 138634 Republic of Singapore; 2 Present Address: A*STAR Skin Research Labs (A*SRL), Agency for Science, Technology and Research (A*STAR), 31 Biopolis Way, #07-01 Nanos, Singapore, 138669 Republic of Singapore; 3 Temasek Life Sciences Laboratory, National University of Singapore, 1 Research Link, Singapore, 117604 Republic of Singapore; 4 Department of Biological Sciences, National University of Singapore, 16 Science Drive 4, Singapore, 117558 Republic of Singapore

**Keywords:** Natural variation in plants, Applied optics, Optical techniques, Biomedical engineering

## Abstract

Traditional methods for assessing plant health often lack the necessary attributes for continuous and non-destructive monitoring. In this pilot study, we present a novel technique utilizing a customized fiber optic probe based on attenuated total reflection Fourier transform infrared spectroscopy (ATR-FTIR) with a contact force control unit for non-invasive and continuous plant health monitoring. We also developed a normalized difference mid-infrared reflectance index through statistical analysis of spectral features, enabling differentiation of drought and age conditions in plants. Our research aims to characterize phytochemicals and plant endogenous status optically, addressing the need for improved analytical measurement methods for in situ plant health assessment. The probe configuration was optimized with a triple-loop tip and a 3 N contact force, allowing sensitive measurements while minimizing leaf damage. By combining polycrystalline and chalcogenide fiber probes, a comprehensive wavenumber range analysis (4000–900 cm^−1^) was achieved. Results revealed significant variations in phytochemical composition among plant species, for example, red spinach with the highest polyphenolic content and green kale with the highest lignin content. Petioles displayed higher lignin and cellulose absorbance values compared to veins. The technique effectively monitored drought stress on potted green bok choy plants in situ, facilitating the quantification of changes in water content, antioxidant activity, lignin, and cellulose levels. This research represents the first demonstration of the potential of fiber optic ATR-FTIR probes for non-invasive and rapid plant health measurements, providing insights into plant health and advancements in quantitative monitoring for indoor farming practices, bioanalytical chemistry, and environmental sciences.

## Introduction

Plants offer a wealth of nutritional benefits, including proteins, vitamins, water, and minerals, as well as various bioactive compounds and secondary metabolites that are known to provide significant health benefits to humans. Research has shown that plant consumption is associated with a lower risk of various diseases such as cancer^1,2^, cardiovascular diseases^3,4^, and diabetes^5,6^. This is due to the presence of phytochemicals, which exhibit various therapeutic properties, including high levels of natural antioxidants that prevent oxidative stress and cell damage. Monitoring plant health is crucial in maintaining the quality and quantity of these beneficial compounds in plants, particularly in agriculture and indoor farming, where it plays an integral role in ensuring sustainable food supply and production. By monitoring plant health, farmers and researchers can optimize growing conditions, prevent disease spread, and maximize food production efficiency^7^ which is especially important in indoor farming, where high-quality crops and resource efficiency are key goals.

Current methods to identify phytochemicals in plants are mostly chemical-based assays that utilize extraction and chromatographic techniques. An example is the Folin–Ciocalteu (F–C) reaction assay which measures the total phenolic content in plant extracts and biological samples based on electron transfer and chemical reduction of a reagent^8^. 2,2-diphenyl-1-picryl-hydrazine-hydrate (DPPH) assay has been extensively researched as an inexpensive analytical tool to measure antioxidant activities in plant extracts^9^. High-performance liquid chromatography (HPLC) is also widely used for isolating and identifying bio-chemicals for quantitative analysis^10^. Over the years, numerous research has started combining multiple detection methods to improve the efficiency of phytochemical analysis^11,12^. However, these methods require biochemical techniques that destroy samples during extraction. The preparation steps and experimental procedures required are also time-consuming and labor-intensive which are not suitable for rapid testing of phytochemicals in agricultural fields and indoor farms.

With the advancement in rapid and non-invasive spectroscopic technology, limitations faced by using wet chemistry methods can be greatly alleviated. Infrared spectroscopy is a vibrational spectroscopy technique that can detect chemical bonds in the samples. In recent years, near-infrared (NIR) and mid-infrared (MIR) spectroscopy have been successfully used for quantifying chemical constituents in various food and plants^13^. In addition, Fourier transform infrared (FTIR) spectroscopy has become an increasingly popular technique to identify functional groups of chemical constituents due to its high signal-to-noise ratio (SNR), fast and real-time spectral collection, and easy usability with minimal to no sample preparation^14^. It has been included in research studies consisting of vast applications such as screening food fraud^15^, analyzing cell wall composition^16^, floral species identification^17^, analysis of bioactive compounds, and antioxidant analysis in food^18–22^ and medicinal plants^23,24^. An attenuated total reflectance (ATR) sampling technique is commonly equipped in combination with FTIR for data acquisition by placing the sample on a high refractive index crystal surface where the infrared beam is completely reflected internally. Evanescent waves with adjustable penetration depth depending on the ATR crystal will be absorbed by the sample and be detected as a change in reflectance at the surface of the crystal. Hence, close contact between the ATR crystal and the sample must be ensured^25^. For in situ spectral analysis, ATR-FTIR fiber probes are used where the incident beam is guided to the ATR crystal using a flexible fiber optic system. To date, in situ ATR-FTIR spectroscopy has mostly been experimented on aqueous solution samples with a few studies focusing on monitoring bioactive compounds in plants^16–18^.

In this pilot investigation, we demonstrate the use of fiber optic ATR-FTIR probes with a customized contact force control unit as a non-invasive and in situ measurement technique to accurately analyze phytochemicals in plants. A series of experiments were conducted on different vegetable leaves with various probe configurations to investigate the sensitivity in detecting phytochemicals in situ. We showed that by combining the FTIR spectra acquired using polycrystalline infrared (PIR) and chalcogenide infrared (CIR) along with the suitable choice of probe type and measuring force, we were able to obtain a sensitive measurement in a broadband wavenumber range with minimal damage on the leaves. The ATR-FTIR spectra collected contain peaks corresponding to the vibrational frequencies of various functional groups present in the leaf, which was used to identify and compare the types of phytochemicals in different vegetables and plants. Additionally, we conducted in situ monitoring of water levels, antioxidant activities, lignin, and cellulose contents in green bok choy plants and calculated the normalized differential indices to observe the impact of ageing and drought stress. Overall, this study presents an innovative approach to studying plant biochemical compositions in a rapid, non-destructive, and in situ manner, advancing the use of optical spectroscopic techniques in plant research.

## Materials and methodology

### Preparation of samples

In the first set of experiments for optimizing the probe configuration and contact force, vegetable samples purchased from the local grocery store were used. The samples included red and green oak leaf lettuce, Chinese red and green spinach, and organic curly kale. Leaves and petioles were randomly selected from each purchased package.

For the second set of experiments, the optimized fiber probe was used to carry out in situ measurements on bok choy plants. Bok choy seeds of a green cultivar (*Brassica rapa subsp. Chinensis*) were surfaced sterilized using a 10% bleach solution containing 0.1% Triton X-100, and then plated on Murashige and Skoog (MS) basal media with 1% agar. Plants were germinated and grown in a growth chamber at 22 °C under 24 h continuous light and 90 µmol m^−2^ s^−1^ light intensity for 4 days. Following this, bok choy seedlings were transferred to soil containing a 10:1 ratio of BVB peat moss (BVB substrates, Netherlands) and sand. Plants were grown at 22 °C under a 16 h light/ 8 h dark cycle, ∼150 μmol m^−2^ s^−1^ light intensity, and 70% relative humidity. Plants were kept well-watered prior to drought treatment. On day 28, plants were soaked with water overnight to ensure saturation of the soil. This was regarded as pretreatment Day 0. On pretreatment Day 0, plants were randomly split into two even groups for control and drought treatments. During treatment day 1, drought treatment began by withholding water. The control group continued to receive regular watering for the rest of the experiment. The measurements were carried out on pre-treatment Day 0 (28-day old plants), treatment Day 8 (36-day old plants), and treatment Day 13 (41-day old plants). All experiments were performed in accordance with relevant institutional guidelines and regulations.

### Experiment methodology

**Figure 1:**
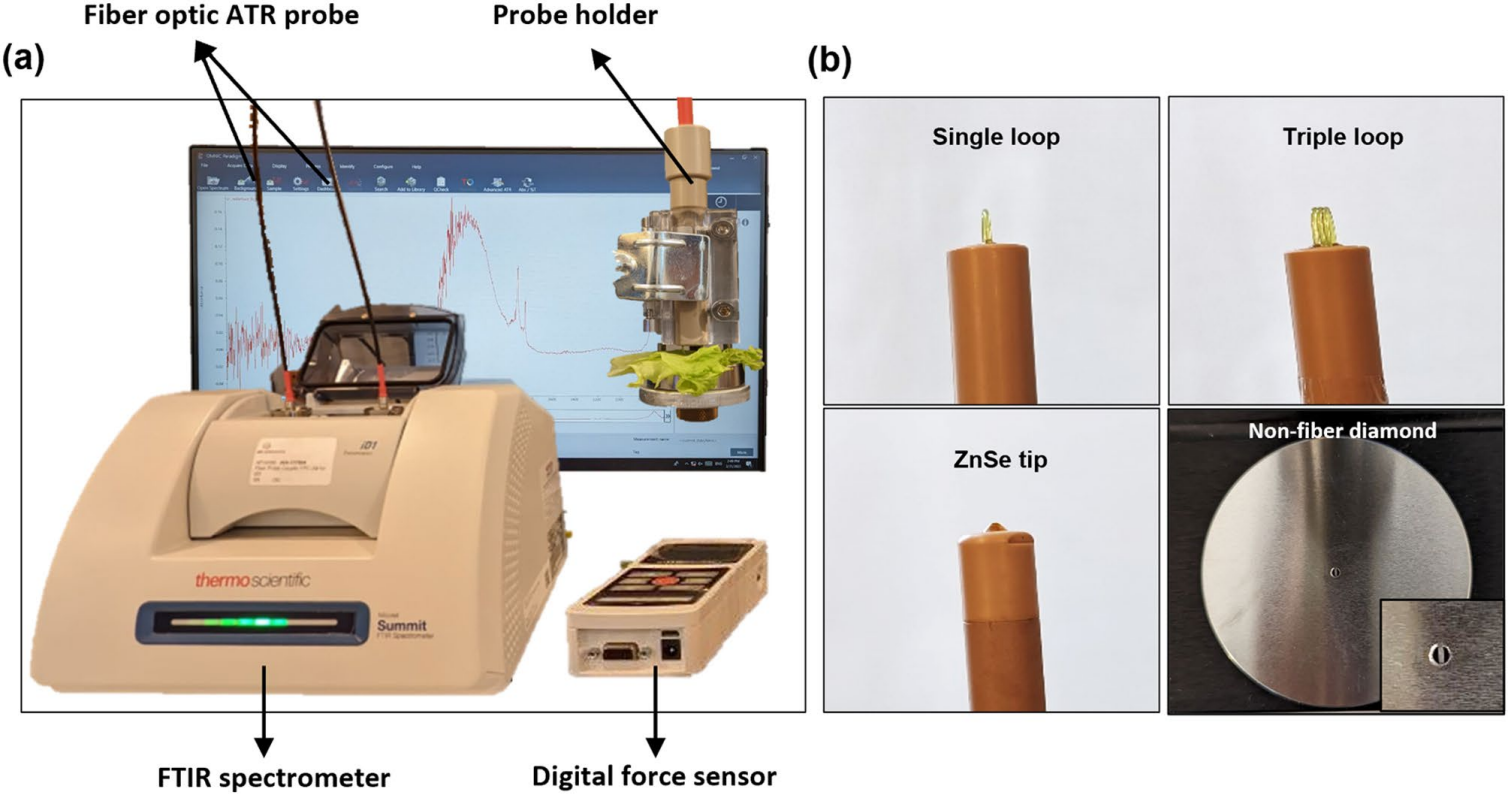
(**a**) Photograph of the experimental set-up which includes an FTIR spectrometer, a fiber optic ATR probe, and an adjustable probe holder connected to a digital force sensor. (**b**) Photographs of four different ATR accessories including single loop, triple loop, ZnSe tip and non-fiber ATR crystal plate (inset shows the diamond crystal that lies in the center of the plate).

The main instrument was a compact FTIR spectrometer equipped with the Everest diamond ATR accessory and a fiber optic accessory (Nicolet Summit, Thermo Fisher Scientific Inc.), as shown in Fig. 1a. The non-fiber diamond ATR accessory has a transmission range of 4000–600 cm^−1^. The fiber-optic ATR probes (FlexiSpec, art photonics GmbH) include a PIR silver halide fiber with a ZnSe tip and a transmission range of 3100–600 cm^−1^, a PIR silver halide fiber with a detachable single or triple loop and a transmission range of 2500–600 cm^−1^, and a CIR chalcogenide glass fiber with a detachable single or triple loop and a transmission range of 6500–1550 cm^−1^, as shown in Fig. 1b. Because the ATR-FTIR signal intensity is sensitive to the applied force, a customized probe holder was fabricated to produce sensitive and reliable readings. It consists of an adjustable contact plate and a digital force sensor to control the contact force between the leaf and the probe precisely. The force sensor contains an advanced force indicator (M5I, MARK-10) and a compression sensor head (MR02-100, MARK-10). The accuracy is up to two decimal points. Absorption spectra were collected with an average of 64 sample scans and a resolution of 4 cm^−1^ using the OMNIC paradigm software. The raw spectra were pre-processed using the asymmetric least squares baseline correction, followed by a Savitzky–Golay filter of second polynomial order with 57 smoothing points. The spectral information acquired from the fiber optic ATR probes is a fusion of the pre-processed CIR wavenumber range (4500–2000 cm^−1^) and PIR wavenumber range (2000–900 cm^−1^).

For the first set of experiments, measurements were performed immediately after purchase to ensure freshness. Due to the large number of instrument configurations, leaf positions (veins and petioles), and vegetable types that needed to be tested on the same day, one measurement was taken per leaf position. For the second set of experiments, measurements were taken on the midrib of each leaf of the plants. On pretreatment Day 0, when no drought had been induced and all the plants were grown under the same conditions, one measurement on the control and one drought plant were performed, resulting in a total of 6 measurements. On treatment Day 8 and treatment Day 13, all plants were measured, and 18 measurements were performed on each day. In total, 42 measurements were performed throughout the course of the experiment. One measurement was determined to be the outlier and removed from analysis because the standard score (Z-score) value was greater than 2 for both PIR and CIR data.

### Normalized index of FTIR peaks

The peak heights at 1022, 1054, 1100, 1148, 1354, 1417, 1634, 1736, 2917, and 3350 cm^−1^, with a bandwidth of 20 cm^−1^, were extracted from preprocessed FTIR spectra. Normalized difference midinfrared reflectance index (NDMRI) values were then calculated for all possible pairs of these peaks using the formula:

$$NDMRI= \frac{peak \;height \;i-peak \;height \;j}{peak \;height \;i+peak \;height \;j}$$

To classify between control and drought groups or among different leaf ages (Day 0, 8, and 13), the spectral features that contain original peak heights and normalized index values were ranked based on ANOVA F-value scores. Spectral features with a high correlation (Pearson’s correlation coefficients greater than 0.95 or lower than − 0.95) were removed. The remaining spectral features were used for further analysis.

## Results and discussion

### Investigation of different ATR-FTIR accessories

**Figure 2:**
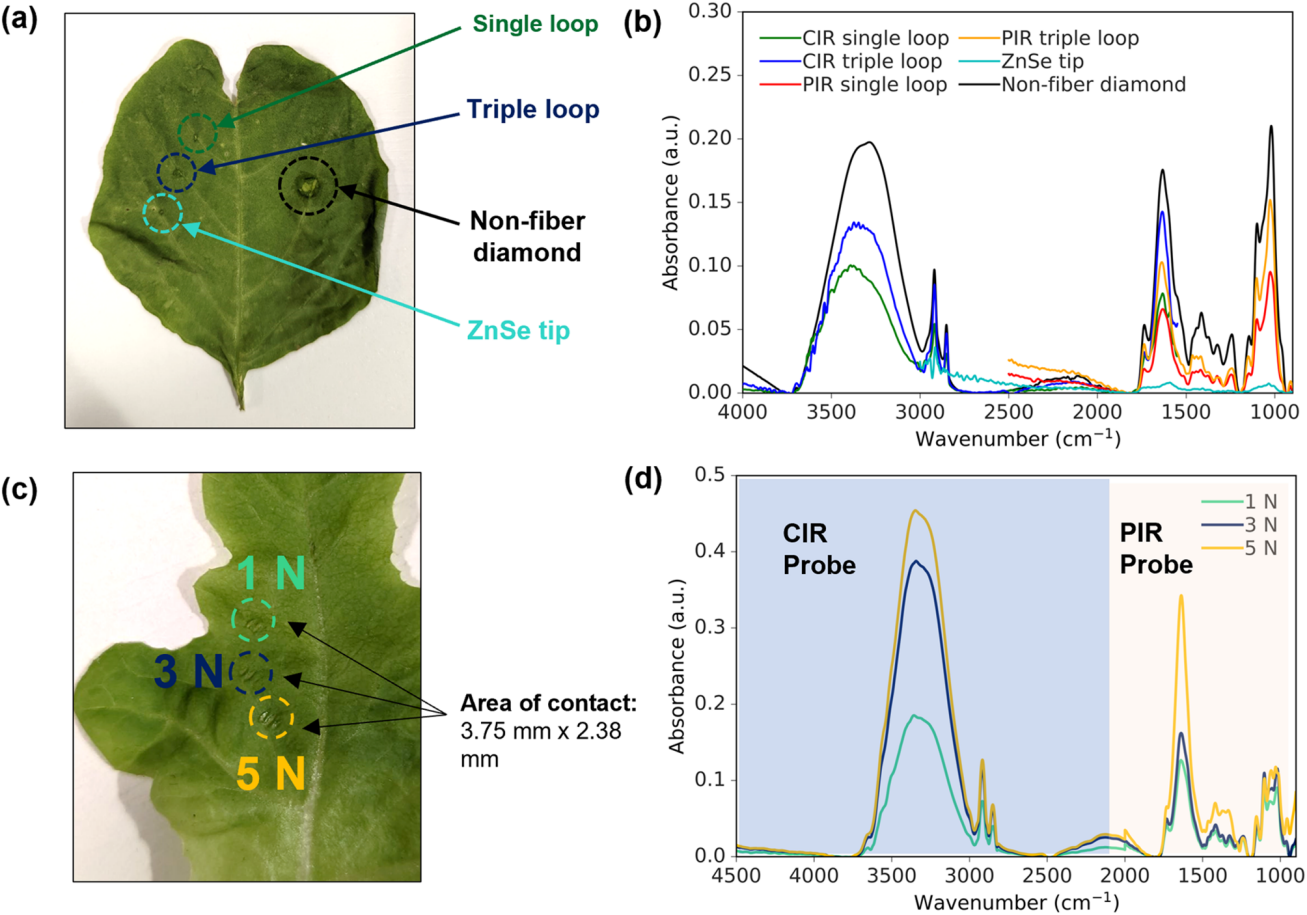
(**a**) Stress marks on a green spinach leaf after the measurements using the different probes. (**b**) Infrared absorption spectra in the range of 4000–900 cm^−1^ using a CIR single loop (green curve), a CIR triple loop (blue curve), a PIR single loop (red curve), a PIR triple loop (orange curve), a ZnSe tip (cyan curve), and a non-fiber diamond ATR (black curve). (**c**) Stress marks on a green lettuce leaf after measurements using 1 N, 3 N and 5 N force respectively. (**d**) Combined infrared spectra of CIR and PIR triple loop measurements of the leaf with a contact force of 1 N (green curve), 3 N (blue curve) and 5 N (orange curve). The CIR region is marked with blue background and the PIR region is marked with light yellow background.

A series of experiments were carried out on leafy vegetables to evaluate the performance of different types of ATR-FTIR accessories. The non-fiber diamond ATR was chosen as the reference standard for comparison because it is the most widely used ATR sampling technique. However, this method requires cutting the leaf from the plant, which makes it unsuitable for remote sensing and in situ measurements. Fiber optic ATR probes are a more flexible and lightweight alternative that allows for continuous and remote monitoring. Figure 2a depicts the stress indentations left on a green spinach leaf after measurements using the various probes. It can be observed that fiber-optic ATR probes generally produce minimal damage to the leaf whereas the non-fiber diamond ATR creates a more noticeable stress mark indicating larger damage. Figure 2b shows the spectrum collected by six types of ATR accessories on the veins of the leaves. The non-fiber diamond ATR provides the broadest wavenumber range and the highest absorbance signal intensity because the pressure clamp produces a fixed contact force which penetrates deep into the leaf but creates greater damage at the same time. Meanwhile, the fiber-optic CIR and PIR single and triple loops produced comparable absorbance signal intensity with a high spectral resolution, but less damage to the leaf. The signals from the triple loops were higher than those from the single loops. The ZnSe tip produced the lowest absorbance intensity. This consistent trend was observed in multiple leaves, leading to the selection of the CIR and PIR triple loops for subsequent studies in this paper.

### Investigation of contact forces

The next step was to investigate the ideal contact force to be exerted on the leaves by the triple loop probes when conducting the experiments to ensure optimal non-destructive optical measurement performance. Figure 2c shows the condition of a green lettuce leaf after measurement with 3 different forces – 1 N, 3 N and 5 N. All 3 forces resulted in minimal indentations (contact area of approximately 3.75 mm × 2.38 mm) with 5 N having the most distinct stress mark while 3 N and 1 N leaving slightly similar stress marks. Figure 2d depicts the combined CIR-PIR spectrum for all 3 contact forces. It shows that the signal intensity with a 5 N force is the highest, followed by 3 N and finally 1 N, as expected. The absorption peaks could be resolved in all cases and the results are reproducible on multiple leaves. For the subsequent experiments, a 3 N force was chosen with a balanced consideration of a high SNR and minimal damage to the leaf.

### Molecular fingerprint analysis of various types of vegetables

**Figure 3:**
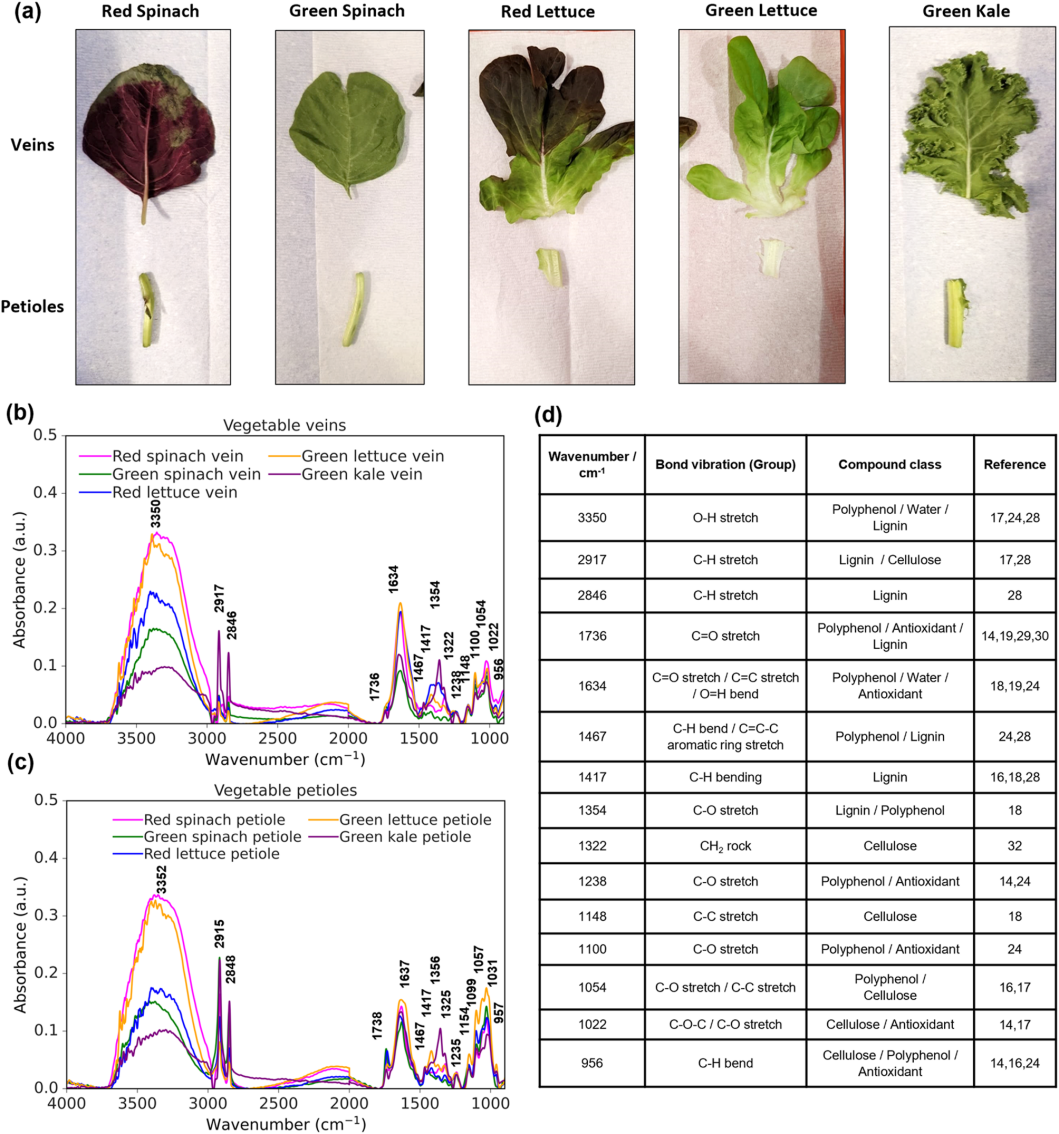
(**a**) Photographs of vegetable leaf samples used which include red and green spinach, red and green lettuce, and green kale leaves. Both the veins and petioles were analyzed. (**b**) FTIR spectra of vegetable veins and (**c**) vegetable petioles using CIR and PIR triple probes with a force applied of 3 N: red spinach (magenta), green spinach (green), red lettuce (blue), green lettuce (yellow), green kale (purple). (**d**) Table showing the wavenumber of the observed FTIR absorbance peaks with the corresponding bond vibrations and chemical information in (**b**) and (**c**).

The infrared spectral features of red and green spinach leaves, red and green lettuce leaves, and green kale leaves were studied, as shown in Fig. 3a. Data were collected at the veins and petioles, as shown in Fig. 3b,c, respectively. The spectral absorption peaks observed between the wavenumber range of 4000–900 cm^−1^ depict the molecular fingerprints that could be used to investigate the chemical compositions of the plants. There are four regions of interpretation which include the hydrogen single bond region (4000–2500 cm^−1^), the triple bond region (2500–2000 cm^−1^), the double bond region (2000–1500 cm^−1^), and the fingerprint region (1500–900 cm^−1^)^16,25^. Figure 3d displays a table that presents the wavenumbers for FTIR absorption features identified in Fig. 3b,c along with the corresponding functional groups and chemical components. Enlarged spectra view of the PIR region is provided in the Supplementary Information (Figure S1) for better examination of spectral characteristics.

Antioxidants refer to substances that can slow down or prevent damage to cells caused by reactive oxygen and other free radicals. In FTIR spectroscopy, the degree of reduction of peaks related to a specific functional group can be utilized to measure antioxidant activity. Polyphenols, such as phenolic acids, flavonoids, lignans, and stilbenes, are secondary metabolites found in fruits and vegetables that possess high antioxidant properties due to the presence of the hydroxyl (O–H) functional group which acts as a hydrogen donor that can react with reactive free radicals. Polyphenolic compounds are composed of an aromatic ring that bears one or more hydroxyl groups. FTIR is a sensitive tool that can identify polyphenols as it can easily reveal characteristic fingerprint bands, which are sensitive to hetero-nuclear functional group vibrations and polar bonds. Referring to the single bond region of the FTIR spectra, the broad and prominent absorption peak from 3700 to 3000 cm^−1^ depicts the O–H stretch bond, most likely contributed by water absorption^17^. For the double bond region, the C=O stretch from esters or phenolic acids can be seen from 1800 to 1600 cm^−1^^19^. Finally, in the fingerprint region, peaks from 1500 to 1400 cm^−1^ are due to the C=C–C stretch vibrations in aromatic rings and C–O stretch can be observed from regions between 1400 to 1300 cm^−1^ and 1000 to 900 cm^−1^^18,24^. The combination of all these functional groups indicates the total polyphenolic content in vegetables. In Fig. 3b, red spinach has the strongest absorbance signal in the O–H stretch region compared to the other vegetables and a relatively high signal in C=O and C–O stretch bonds. On the other hand, green kale exhibits weak signals in the O–H stretch and C=O stretch bonds. This implies that the polyphenol content in red spinach is likely to be the highest, while it is the lowest in green kale. This is consistent with studies that utilized various chromatographic and spectrometric techniques to quantify polyphenolic content in spinach and kale^26,27^.

Besides polyphenolic contents, lignin can also be analyzed from the FTIR spectrum. It is a complex phenolic macromolecule that provides structural support and thickening to the cell walls of plants. It is mainly composed of many crosslinked monolignol units which contain several functional groups such as hydroxyl and methoxy (O-CH3) attached to an aromatic ring. The region between 2950 to 2850 cm^−1^ is assigned to the C-H stretch from aliphatic methyl groups^28,29^. In Fig. 3b, the absorbance signal for green kale is the strongest in this region compared to the other vegetables. In the fingerprint region, kale also has the most intense signal at 1467 cm^−1^ and 1354 cm^−1^ associated with the C–H bend and C–O stretch group, respectively^18,28,30^. The higher contents of aliphatic compounds and methyl groups in kale suggest a higher lignin content. This is reasonable because the kale species is a member of the cruciferous vegetable family known for having tough fibrous leaves which usually translate to a higher amount of lignin content as compared to the more delicate leaves of spinach and lettuce^28,31^. The results are reproducible on multiple leaves and pure lignin spectra were also measured separately for reference as shown in Supplementary Information Figure S2.

Cellulose is another crucial component of plant cell walls, comprising chains of glucose molecules linked by beta 1–4 glycosidic bonds. These bonds are responsible for the strong and rigid structure of cellulose and can be identified by the characteristic absorption band at 1022 cm^−1^ representing the C–O–C stretching vibrations^17^. In addition to this peak, several other absorption bands can typically be observed in the FTIR spectrum of cellulose, corresponding to the vibrational modes of its various functional groups. These bands include the O–H stretching bond at 3700–3000 cm^−1^, the C–H stretching vibration at 2917 cm^−1^, C–C stretching vibrations in the range of 1150–1050 cm^−1^, and a slight shoulder can also be observed at 1322 cm^−1^ attributed to the CH_2_ rocking vibration^17,18,32^. Pure cellulose spectra were also measured separately for reference as shown in Supplementary Information Figure S2.

Figure 3c shows that similar and consistent observations can be made for the measurements of vegetable petioles compared to the veins. The results indicate that red spinach leaves have the highest signal intensity for polyphenol contents, while green kale leaves exhibit the weakest signal intensity in the wavenumber ranges of 3700–3000 cm^−1^, 1800–1600 cm^−1^, 1500–1300 cm^−1^, and 1000–900 cm^−1^. Additionally, the green kale leaves also exhibit the highest absorbance signal representing lignin compared to the other vegetables, as evidenced by absorption peaks at wavenumber ranges of 2950–2850 cm^−1^ and 1500–1300 cm^−1^. Moreover, when comparing petioles with veins, the absorbance peaks of the C–H stretching vibrations of petioles are relatively higher than those of veins. In addition, the cellulose peaks that are prominently displayed in the spectra exhibit higher absorbance signals in petioles than in veins. This could be attributed to the fact that petioles are typically thicker in structure, and therefore contain a greater number of structural components such as cellulose.

### In situ monitoring of plants

**Figure 4:**
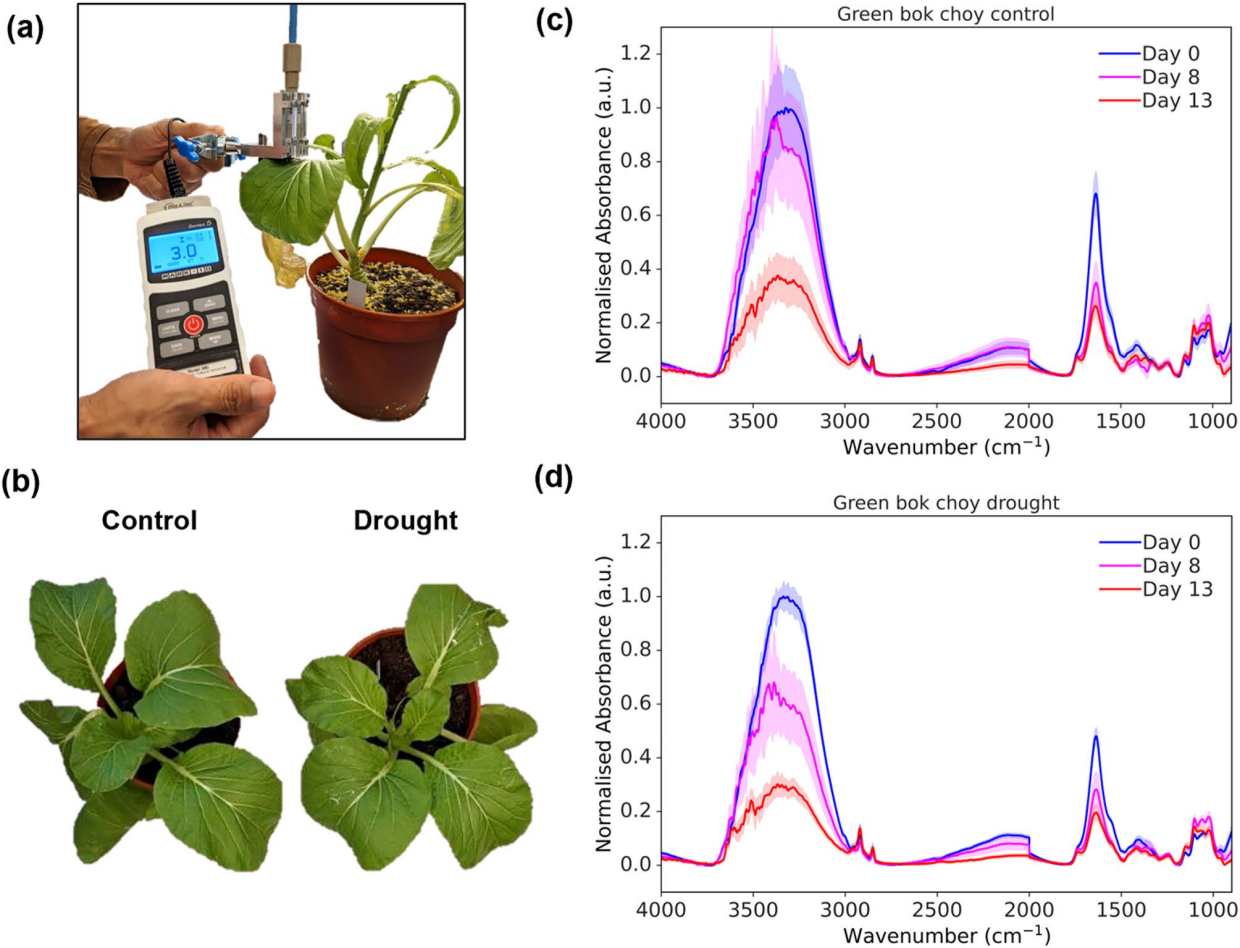
(**a**) In situ measurement of the green bok choy plants under drought stress using the fiber optic ATR probe. The total sample size consisted of 42 measurements with 1 outlier excluded from plots. (**b**) Pictures of control and drought plants on treatment Day 8. (**c**) Normalized FTIR spectrum for the control group and (**d**) drought group on pretreatment Day 0 (blue), treatment Day 8 (magenta) and treatment Day 13 (red). The mean values are plotted as solid lines and the standard deviations are plotted as shaded areas.

Finally, we demonstrated the use of the fiber optic ATR probe to perform non-invasive and in situ monitoring of potted plants under drought stress over a period of 13 days. As shown in Fig. 4a, the measurement setup involved clamping the probe onto the leaves. The contact force was monitored using the force sensor. The midribs of the leaves were selected for measurement as they were found to provide the most effective absorbance signal compared to other parts of the leaves, see Supplementary Information Figure S3. On treatment Day 8, the drought group of plants displayed a slight yellowing of leaves, which is a visible sign of drought stress, as shown in Fig. 4b. In addition, the drought group of plants showed reduced leaf area (Supplementary Information Figure S4a) and chlorophyll content (Supplementary Information Figure S4b), consistent with physical signs of drought stress. Additional images of all the plants used in the experiment, including both control and treated groups, can be found in Supplementary Figure S4c. The normalized absorbance spectra of the control group and drought group on Day 0, Day 8, and Day 13 are shown in Fig. 4c,d, respectively. All preprocessed data was normalized by dividing it with the mean absorption value observed on Day 0 at 3300 cm^−1^ where the maximum absorption was recorded, allowing for a fair and quantitative comparison of changes over time. In both cases, a noticeable decrease in absorbance across the significant FTIR peaks of water (3700–3000 cm^−1^ and 1750–1500 cm^−1^), accompanied by an increase in the lignin (2900–2800 cm^−1^, 1800–900 cm^−1^) and cellulose (2900–2800 cm^−1^, 1630–900 cm^−1^) contents were observed. The decrease in water peaks was expected due to water loss, which was observed to accelerate from Day 8 to Day 13. The increase in the lignin and cellulose could be attributed to the production of these chemicals during drought stress to strengthen the cell walls and reduce water loss, demonstrating the plants’ ability to protect themselves and cope with the stressful environment^33–35^.

**Figure 5:**
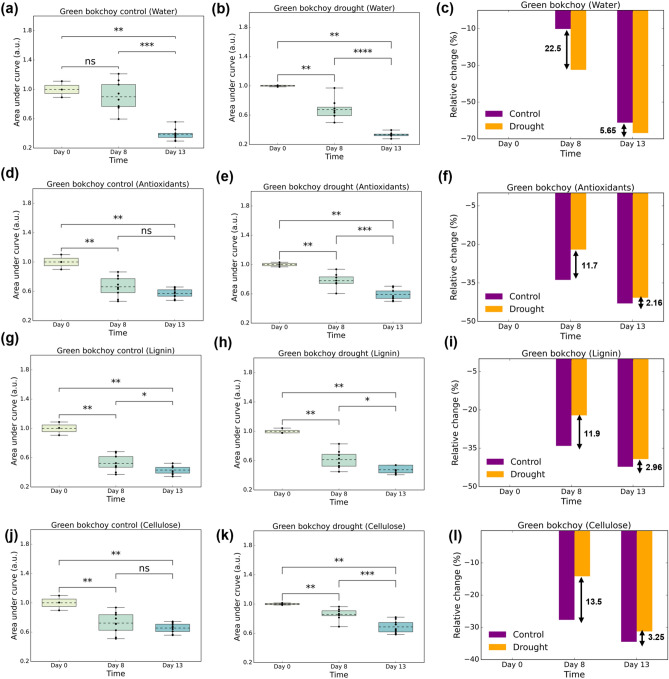
(**a**,**b**) Box plots of the area under the curve for water peaks at wavenumber ranges of 3700–3000 cm^−1^ and 1750–1500 cm^−1^ for the control (purple) and drought (yellow) groups, respectively with (**c**) the relative percentage change in content with reference to Day 0. (**d**,**e**) Box plots of the area under the curve for antioxidant peaks at wavenumber ranges of 1800–900 cm^−1^ for the control and drought groups, respectively with (**f**) the relative percentage change in content with reference to Day 0. (**g**,**h**) Box plots of the area under the curve for lignin peaks at wavenumber ranges of 2900–2800 cm^−1^ and 1800–900 cm^−1^ for the control and drought groups, respectively with (**i**) the relative percentage change in content with reference to Day 0. (**j**,**k**) Box plots of the area under the curve for cellulose peaks at wavenumber ranges of 2900–2800 cm^−1^ and 1630–900 cm^−1^ for the control and drought groups, respectively with (**l**) the relative percentage change in content with reference to Day 0.

To assess the quantitative changes, we calculated the area under the curve and the relative percentage changes for specific wavenumber ranges associated with corresponding biochemicals. The selection of wavenumber ranges was based on literature and validated by measuring the FTIR spectra of pure chemical compounds (Figure S2). The wavenumber ranges chosen were 3700–3000 cm^−1^ and 1750–1500 cm^−1^ for the water content^27^, 1800–900 cm^−1^ for the antioxidant content^18^, 2900–2800 cm^−1^ and 1800–900 cm^−1^ for the lignin content^28^, and 2900–2800 cm^−1^ and 1630–900 cm^−1^ for the cellulose content^36^. The selected wavenumber ranges correspond to the numerous functional groups present in each of the compounds being analyzed. The combinations of wavenumber ranges provide a clearer picture of the biochemical contents of plant leaves being measured. The analysis for the area under the curve could be used to compare the changes across different days for the same group, whereas the analysis for the relative percentage change could be used to compare the changes for different groups on the same day. The subsequent analysis results are discussed for water, antioxidants, lignin and cellulose. Due to the overlapping of the O–H group in polyphenols with the absorption spectrum of water, it can be difficult to accurately measure polyphenol levels using this technique. As a result, this study shifted its focus to examining the levels of antioxidants, which rely on other functional groups to be distinguished rather than the O–H group. We computed the areas under the curves by integrating the absorbance values within the specified wavenumber ranges for both the control group (Fig. 5a,d,g,j) and the drought group (Fig. 5b,e,h,k). Subsequently, we normalized the calculated areas for each daily sample within each group to enhance the clarity of data trends and to facilitate meaningful comparisons across various days and groups. Additionally, we computed the relative percentage change by subtracting the absorbance values on treatment Day 8 and treatment Day 13 from those recorded on pretreatment Day 0, as depicted in Figs. 5c,f,i,l. For the control group, Fig. 5a shows a significant change between Day 0 and Day 13 (*p*-value: 4.545 × 10^−3^) while no significant changes in water content were observed between Day 0 and Day 8. This was expected since the plants were sufficiently watered during the experiment, and significant changes were eventually observed on Day 13, possibly due to ageing. For the drought group (Fig. 5b), significant changes in water content were observed between Day 0 and Day 8 (*p*-value: 6.061 × 10^−3^) as well as between Day 0 and Day 13 (*p*-value: 4.545 × 10^−3^). With regards to the relative percentage change, both the control and drought groups experienced a gradual decrease in water content over the 13-day period (Fig. 5c), which is a natural occurrence as the plant ages^37^. This decline in water content with age is typically observed as a plant develops, reflecting ongoing physiological changes and metabolic processes^37^. However, the drought group displayed a more pronounced reduction in water content compared to the control group, with a relative change of 22.5% and 5.65% more on Day 8 and Day 13, respectively. This notable decline can be attributed to the added drought stress which causes greater water loss in plants due to hampering of plant growth and hence inhibition of plant nutrients^38^. On the 8th day, plants in drought group may be experiencing a more acute stress response, resulting in a greater relative change in water content. However, by the 13th day, the plants may have undergone adaptive changes, such as the increase in mechanical strength of tracheary elements, which enhances their resilience to water stress. This adaptation likely contributes to the smaller relative change difference in water content on the 13th day between control and drought group.

The analysis of the antioxidant content revealed that the control group (Fig. 5d) showed similar significant changes between Day 0 and Day 8 (*p*-value: 4.545 × 10^−3^) and between Day 0 and Day 13 (*p*-value: 4.545 × 10^−3^). The drought group (Fig. 5e) exhibited similar significant change between Day 0 and Day 8 (*p*-value: 6.061 × 10^−3^) and between Day 0 and Day 13 (*p*-value: 4.545 × 10^−3^). Both control and drought groups showed a decrease in the antioxidant content over the 13-day period, as illustrated in Fig. 5f. The drought group exhibited a smaller relative change in antioxidant content compared to the control group, with a relative change of 11.7% and 2.16% less on Day 8 and Day 13, respectively. When plants undergo drought stress, there is excessive production of reactive oxygen species (ROS) in response to water shortages. Thus, our results suggest that the reduction in antioxidant content is comparatively lower in drought-treated plants due to the plant’s adaptive response to stress by increasing the production of antioxidants to protect against oxidative damage caused by ROS^38^.

The control group’s lignin content (Fig. 5g) exhibited similar significant changes between Day 0 and Day 8 (*p*-value: 4.545 × 10^−3^) and between Day 0 and Day 13 (*p*-value: 4.545 × 10^−3^). The drought group (Fig. 5h) also showed similar significant changes between Day 0 and Day 8 (*p*-value: 6.061 × 10^−3^) and between Day 0 and Day 13 (*p*-value: 4.545 × 10^−3^). As shown in Fig. 5i, both the control and drought groups experienced a reduction in lignin content over the 13-day period, but the drought group had a smaller relative change than the control group, with a decrease of 11.9% and 2.96% on Day 8 and Day 13, respectively. Moreover, the cellulose content in the control group (Fig. 5j) displayed similar significant changes between Day 0 and Day 8 (*p*-value: 9.091 × 10^−3^) and between Day 0 and Day 13 (*p*-value: 4.545 × 10^−3^). The drought group (Fig. 5k) exhibited similar significant changes between Day 0 and Day 8 (*p*-value: 6.061 × 10^−3^) and between Day 0 and Day 13 (*p*-value: 4.545 × 10^−3^). Figure 5l shows that the cellulose content decreased in both groups, with smaller relative changes in the drought group compared to the control group at 13.5% and 3.25% on Day 8 and Day 13, respectively. Both lignin and cellulose are critical components of the plant cell wall, and as the plant ages, cell wall composition changes and can result in a reduction in lignin and cellulose content. However, when subjected to drought stress, plants adapt by increasing the synthesis of lignin and cellulose to improve cell wall strength, thereby helping it maintain structural integrity when water is scarce, as suggested by previous studies^39–42^.

### Normalized difference reflectance index of FTIR Peaks

**Figure 6:**
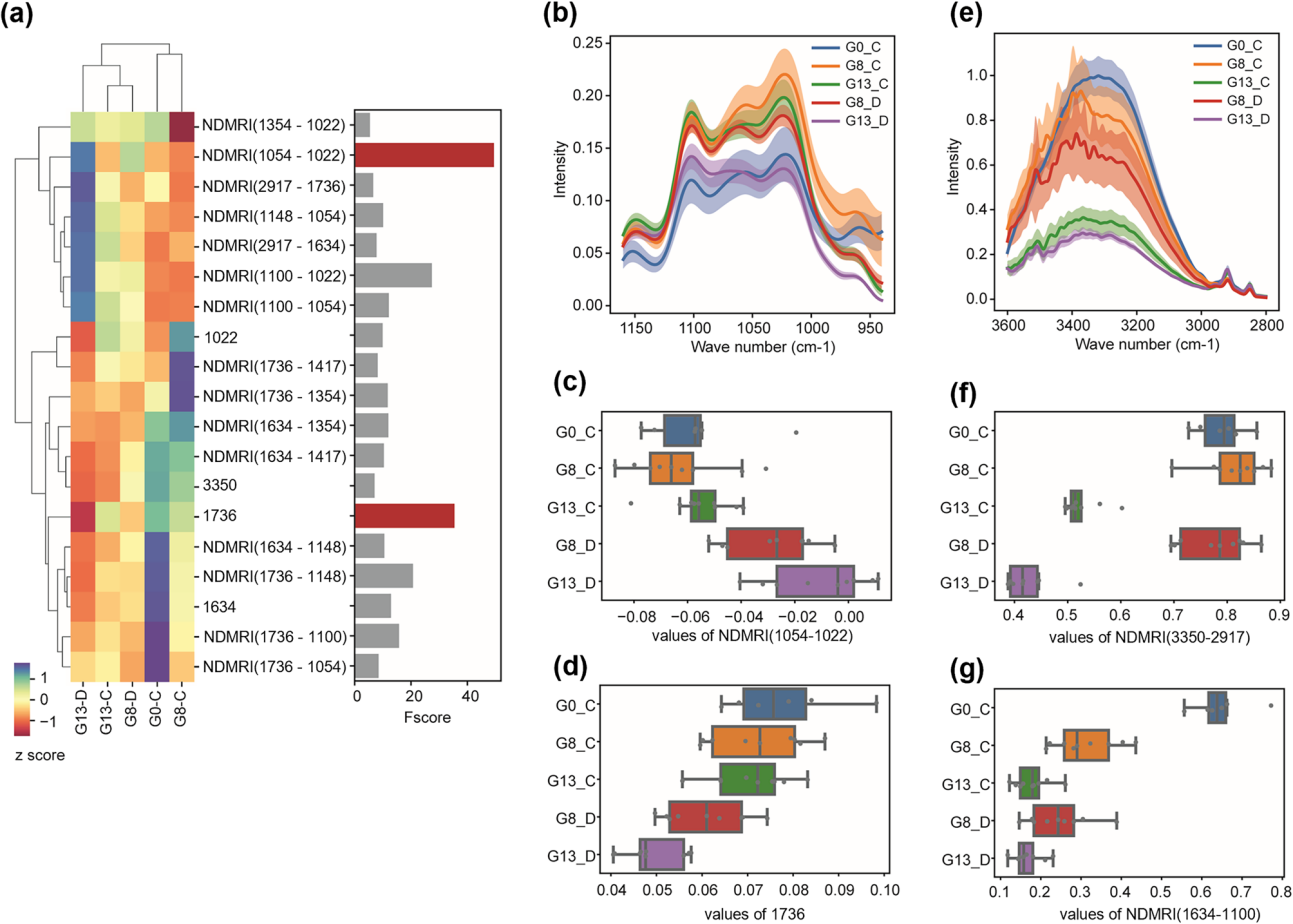
FTIR peaks related with drought stress and leaf age. (**a**) Heatmap shows Z score values of peak height and normalized peak index across different sample groups, with the bar graph on the right displaying the ANOVA F value scores for classification between drought and control groups. The top two features are highlighted in red. Note that only features with F value score greater than 5 are shown. The average linkage method with Euclidean distance was used to obtain the dendrograms. N = 6 spectral data per group. (**b**,**e**) FTIR spectra relevant to the top features obtained from control versus drought comparison (**b**) and leaf age comparison (**e**). The graphs show the mean values with 95% confidence intervals. (**c**,**d**,**f**,**g**) Boxplots showing values of top two features from control versus drought comparison (**c**,**d**) and leaf age comparison (**f**,**g**). Boxes show 25th, 50th and 75th percentile values, while whiskers show 1.5 interquartile ranges.

Principal Component Analysis (PCA) was performed to show the distribution of data (Supplementary Information Figure S5) and normalized differential analysis of FTIR peaks was conducted in order to identify those linked to drought stress and leaf age. The differential analysis allows us to identify spectral features that are associated with the trait changes of interest, by using an internal reference peak that is independent of those traits. We generated NDMRIs for ten peaks with a peak height greater than 0.05. The average linkage method with Euclidean distance was used to obtain the dendrograms. Our ANOVA F-value scores showed that the peak height at 1736 cm^−1^ and the differential index with 1054 cm^−1^ and 1022 cm^−1^ were highly associated with the comparison between drought and control conditions (Fig. 6a–d). This was due to the greater reduction of 1022 cm^−1^ peak (C–O–C stretching vibrations) under drought conditions compared to the other peak at 1054 cm^−1^ (C–C stretching vibrations). Our ANOVA F-value scores comparing three leaf age groups selected NDMRIs of 3350 cm^−1^ and 2917 cm^−1^ and of 1634 cm^−1^ and 1100 cm^−1^ as the top two spectral features (Fig. 6e–g, Supplementary Information Figure S6). The index of 3350 cm^−1^ and 2917 cm^−1^, water, and lignin peaks respectively, exhibited clear separations of Day 13 data from those at Day 0 and Day 8. Interestingly, the NDMRIs tended to show greater F-value scores compared to those using peak height values, indicating that they are more useful for the classification of plant health status, such as drought versus control comparison.

## Conclusion

In conclusion, this pilot study presents a novel technique for non-invasive and continuous monitoring of plant health using a customized fiber optic-based ATR-FTIR spectroscopy probe. The developed methodology contributes to the field of analytical chemistry by addressing the need for improved chemical measurement science in plant health assessment. The optimized probe configuration and contact forces allowed for capturing FTIR fingerprint features of phytochemicals across a broad wavenumber range (4000–900 cm^−1^), while minimizing damage to plant leaves. The results revealed significant variations in polyphenol content between red spinach and green kale leaves, demonstrating the system’s ability to quantify phytochemical differences among plant species. Furthermore, the system successfully monitored green bok choy plants subjected to induced drought stress over a 13-day period. The findings showed that water content, antioxidant activity, lignin, and cellulose content could be quantified and tracked, providing insights into plant growth and responses to external stresses. The drought group exhibited a pronounced reduction in water content, while the relative changes in antioxidant, lignin, and cellulose contents were smaller, suggesting that plants enhance the synthesis of these components to adapt to environmental stresses. NDMRIs derived from statistical analysis of spectral data have been demonstrated to be an effective indicator for the identification of drought and senescence in plants.

This study represents a pioneering demonstration of an optimized ATR-FTIR system for non-destructive, in situ, and rapid measurements of plants, holding significant potential for advancements in the agricultural field and indoor farming. Further, the presented technique has implications in various fields, including bioanalytical chemistry, environmental sciences, and forensics, and paves the way for further developments in analytical methodologies. While we acknowledge that this method may have its constraints when applied to high throughput analysis involving diverse plant species, its true potential shines in large-scale, in-situ scenarios, especially for selective sampling. Our study highlights the impact of leaf structures, including the petiole, leaf lamina, and leaf vein, on FTIR peak heights and patterns. The selection of specific sampling locations should align with the goals of each project. While leaf veins provide enhanced sensitivity to plant phytochemicals, this sensitivity can introduce high data variations, particularly when compared to leaf lamina data. The number of data collections required to draw reliable conclusions depends on factors such as the absolute distance between compared groups and sample variability. By thoughtfully selecting specific plant types, strategically choosing locations and sample size for spectral data collection, this approach holds promise of uncovering valuable insights into environmentally induced phytochemical changes. In addition, future works should focus on enhancing the depth of the research by incorporating comprehensive biochemical data to complement with the findings derived from the fiber-based ATR-FTIR analysis.

### Supplementary Information

Supplementary Figures.

## Data Availability

Data is available upon request and should be addressed to D.U., U.S.D. or M.O.
