# Supplementary material for: A pilot study on non-invasive in situ detection of phytochemicals and plant endogenous status using fiber optic infrared spectroscopy
Source: Sci Rep. 2023 Dec 14;13:22261. doi: 10.1038/s41598-023-48426-5 (PMC10721643; doi:10.1038/s41598-023-48426-5)
Supplement: Supplementary file 1 — Supplementary Figures. [file 41598_2023_48426_MOESM1_ESM.pdf]

Supporting Information

A Pilot Study on Non-Invasive In Situ Detection of Phytochemicals and Plant Endogenous Status Using Fiber Optic Infrared Spectroscopy

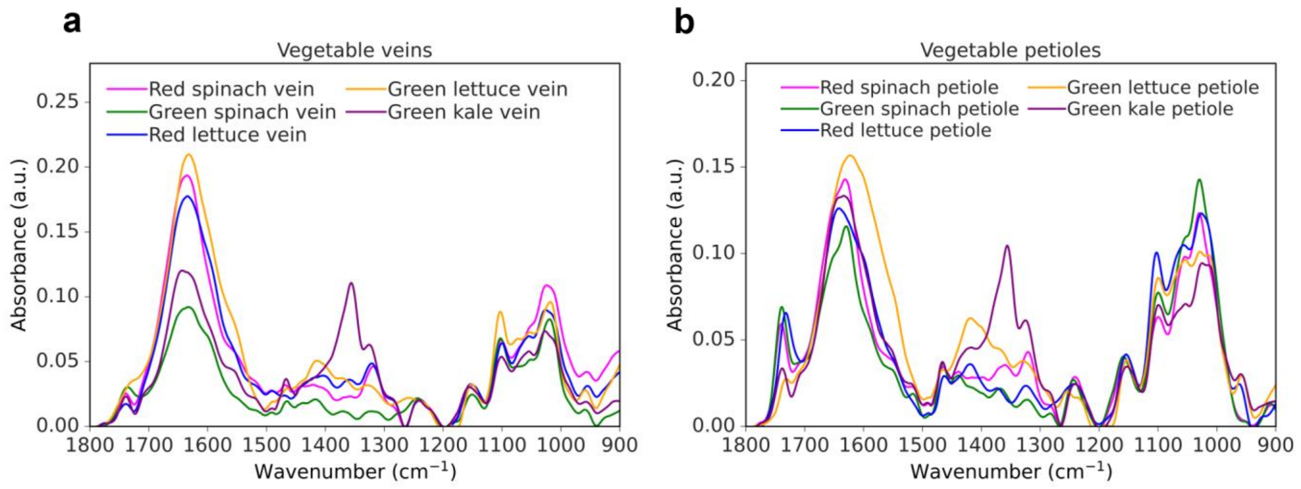

Figure S1. Zoomed in PIR triple loop measurement of vegetable (a) veins and (b) petioles, using an applied force of 3N.

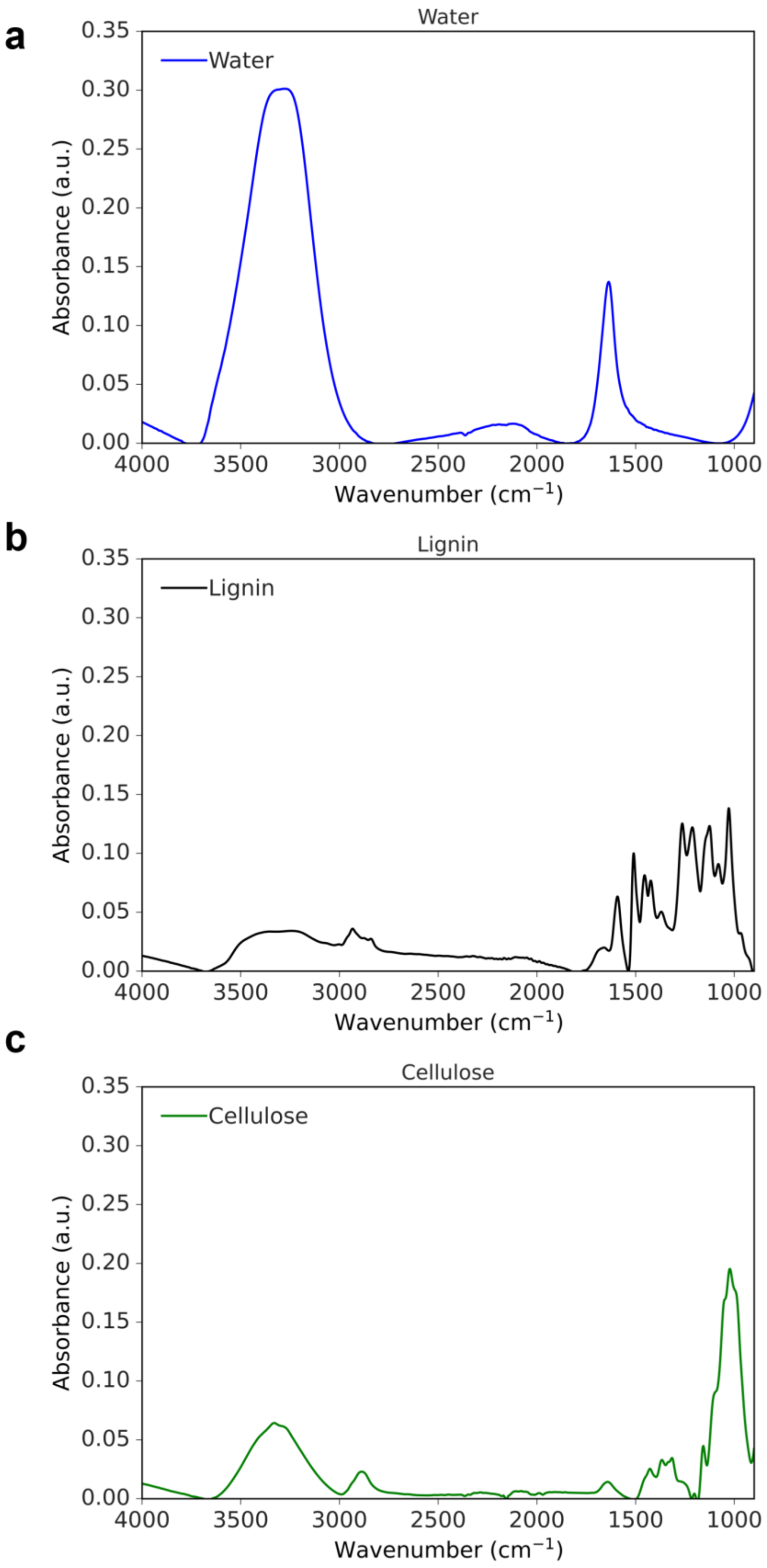

Figure S2. FTIR spectrum extracted using non-fiber diamond ATR for (a) water, (b) pure lignin powder and (c) pure cellulose powder.

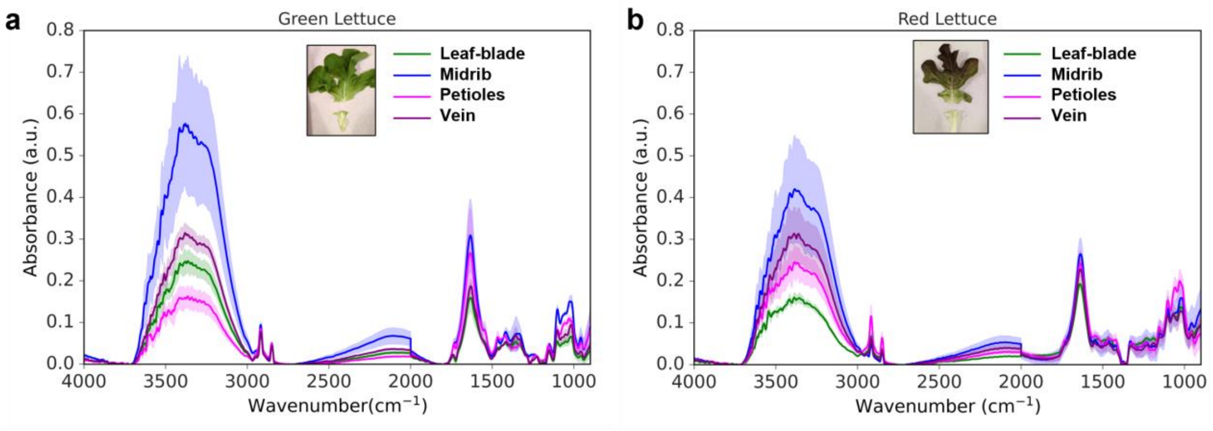

Figure S3. Combined FTIR spectra from CIR-PIR triple loop measurements at four locations of green lettuce (a) or red lettuce (b) – leaf-blade, midrib, petioles, and vein. Measurements were performed with the contact force of 3 N. Solid lines and transparent bands show the mean values and standard deviations from 16 samples for green and red lettuce, respectively.

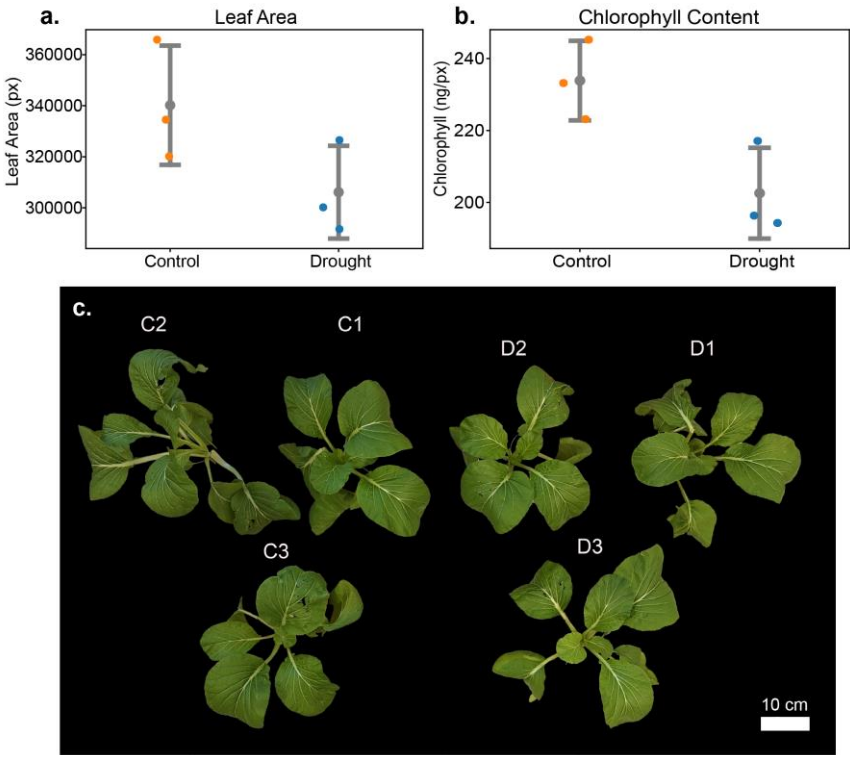

Figure S4. Quantification of chlorophyll content and leaf area in bok choy subject to drought stress. a-b. Leaf area (a) and chlorophyll content (b) of control and drought-treated bok choy plants. Orange dots represent individual plants in the control group, while blue dots represent individual plants in the drought treated group. The error bar represents standard deviation, while the grey dot represents the mean. c. Images of control (C1-C3) and drought (D1-D3) treated plants used in the experiment. Scale bar represents 10 cm.

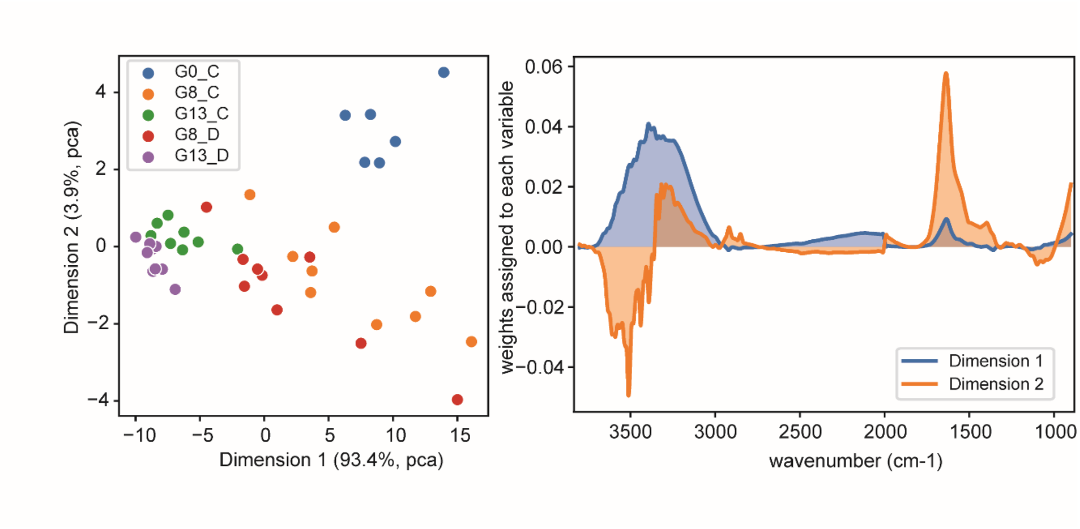

Figure S5. Principal component analysis of FTIR spectra. The scattered plot shows the distribution of collected spectra on the first two axes. The right graph displays the results of factor analysis.

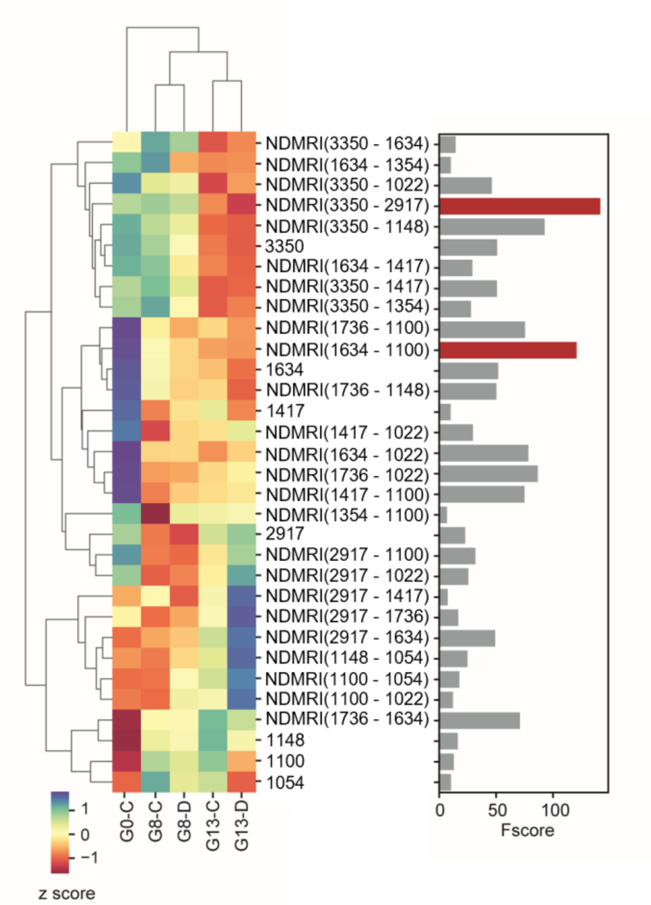

Figure S6. FTIR peaks related with leaf age. (A) Heatmap shows Z score values of peak height and normalized peak index across different sample groups, with the bar graph on the right displaying the ANOVA F value scores for classification between drought and control groups. The top two features are highlighted in red. Note that only features with F value score greater than 5 are shown.
